# Acute kidney injury and infections in patients taking antihypertensive drugs: a self-controlled case series analysis

**DOI:** 10.2147/CLEP.S146757

**Published:** 2018-01-30

**Authors:** Kathryn E Mansfield, Ian J Douglas, Dorothea Nitsch, Sara L Thomas, Liam Smeeth, Laurie A Tomlinson

**Affiliations:** 1 Department of Non-Communicable Disease Epidemiology; 2 Department of Infectious Disease Epidemiology, London School of Hygiene and Tropical Medicine, London, UK

**Keywords:** acute kidney injury, angiotensin-converting enzyme inhibitors, angiotensin receptor antagonists, infection, self-controlled case series

## Abstract

**Background:**

The relative risk of acute kidney injury (AKI) following different infections, and whether angiotensin-converting enzyme inhibitors (ACEIs)/angiotensin receptor blockers (ARBs) modify the risk, is unclear. We aimed to determine the risks of hospital admission with AKI following infections (urinary tract infection [UTI], lower respiratory tract infection [LRTI], and gastroenteritis) among users of antihypertensive drugs.

**Methods:**

We used UK electronic health records from practices contributing to the Clinical Practice Research Datalink linked to the Hospital Episode Statistics database. We identified adults initiating ACEIs/ARBs or alternative antihypertensive therapy (β-blockers, calcium channel blockers, or thiazide diuretics) between April 1997 and March 2014 with at least 1 year of primary care registration prior to first prescription, who had a hospital admission for AKI, and who had a primary care record for incident UTI, LRTI, or gastroenteritis. We used a self-controlled case series design to calculate age-adjusted incidence rate ratios (IRRs) for AKI during risk periods following acute infection relative to noninfected periods (baseline).

**Results:**

We identified 10,219 eligible new users of ACEIs/ARBs or other antihypertensives with an AKI record. Among these, 2,012 had at least one record for a UTI during follow-up, 2,831 had a record for LRTI, and 651 had a record for gastroenteritis. AKI risk was higher following infection than in baseline noninfectious periods. The rate ratio was highest following gastroenteritis: for the period 1–7 days postinfection, the IRR for AKI following gastroenteritis was 43.4 (95% CI=34.0–55.5), compared with 6.0 following LRTI (95% CI=5.0–7.3), and 9.3 following UTI (95% CI=7.8–11.2). Increased risks were similar for different antihypertensives.

**Conclusion:**

Acute infections are associated with substantially increased transient AKI risk among antihypertensive users, with the highest risk after gastroenteritis. The increase in relative risk is not greater among users of ACEIs/ARBs compared with users of other antihypertensives.

## Plain language summary

A sudden decrease in kidney function, known as “acute kidney injury” (AKI), is common and associated with an increased risk of death, prolonged hospital stay, and risk of permanent kidney failure. One of the common causes of AKI is thought to be severe infections, particularly gastroenteritis. However, the degree of increased risk after infections is not known. In addition, some evidence suggests that AKI can occur as a side effect of angiotensin-converting enzyme inhibitors (ACEIs) and angiotensin receptor blockers (ARBs), medications commonly used for treating conditions such as high blood pressure and heart problems. It is thought that AKI risk is particularly increased among people taking these drugs who develop severe infections. Therefore, we examined the relative risk of AKI after urinary tract and chest infections, and gastroenteritis. We also compared the relative risk of AKI after these infections for patients taking a range of blood pressure drugs. Our results show that there is a substantially increased risk of AKI immediately after all three infections, which is particularly marked after gastroenteritis. However, the level of increased risk of AKI after infection was similar in users of ACEIs/ARBs and other blood pressure drugs. Our results suggest that clinicians need to be aware of AKI risk after common infections and that this risk applies to patients taking all classes of antihypertensives.

## Introduction

Acute kidney injury (AKI) is a rapid (within hours or days) deterioration in kidney function, associated with increased mortality,^1^,^2^ prolonged hospital stay,^3^,^4^ and the risk of chronic kidney disease.^5^ AKI has been observed in up to 20% of UK adult hospital admissions.^1^,^6^,^7^ Sepsis and diarrhea leading to hypovolemia are risk factors for AKI, and this is considered to be particularly important for patients taking drugs such as angiotensin-converting enzyme inhibitors (ACEIs) and angiotensin receptor blockers (ARBs).^8^ There is an increased focus on early detection and better management of AKI in primary care.^9^–^12^ However, AKI risk among patients presenting to general practitioners (GPs) with common infections is unknown. In addition, the extent to which this risk is increased among patients prescribed ACEIs/ARBs compared with users of other antihypertensive drugs is unclear. This information is needed to allow health care professionals to identify patients at a high risk of AKI.

We used a self-controlled case series (SCCS) design^13^ to assess the risk of AKI associated with three different types of acute infection: urinary tract infection (UTI), lower respiratory tract infection (LRTI), and gastroenteritis. Using this method, we compared the incidence of AKI during periods of infection compared with that during noninfected (baseline) periods within individual patients. This limits the potential confounding effect of characteristics that vary between individuals, such as comorbidities and risk factors for renal disease.^14^ We also investigated how the relative risk of AKI associated with acute infection differed in ACEI/ARB users compared with the risk in users of other antihypertensives, which are prescribed for similar indications and not considered substantial risk factors for AKI.^8^ In addition, we investigated whether diabetes, cardiac failure, chronic kidney disease, and concurrent prescription of loop diuretics modified the infection-specific increase in the relative risk of AKI.

## Methods

### Study design and setting

We undertook an SCCS study using computerized clinical records from adults attending primary care practices contributing to the UK Clinical Practice Research Datalink (CPRD) and linked hospital record data from the Hospital Episode Statistics (HES) database. CPRD is a database of primary care electronic health record data from 7% of the UK population.^15^ Included patients are largely representative of the UK population.^15^–^17^ HES records cover all admissions for National Health Service (NHS)-funded patients treated either in English NHS trusts or by independent providers.^18^ Seventy-five percent of English general practices included in CPRD are linked to HES data.^15^ The study period was from April 1, 1997, to March 31, 2014. CPRD data are anonymized before being supplied to researchers. Researchers do not have access to names, addresses, or dates of birth (although the year of birth is available). Primary care data are linked with hospital record data by a trusted third party in an approved manner.

### Participants, exposures, and outcomes

We identified all adults (aged ≥18 years) with a first prescription for an ACEI/ARB or other antihypertensive (β-blocker, calcium channel blocker [CCB], or thiazide diuretic) between April 1997 and March 2014, who had a hospital admission for AKI and a primary care record for incident UTI, LRTI, or gastroenteritis.

To ensure that we had reliable measures of drug use and incident morbidity, we required that all participants had at least 1-year continuous registration in CPRD before the first-recorded antihypertensive prescription (ACEI/ARB, β-blocker, CCB, or thiazide diuretic) and that they be registered with a practice meeting CPRD’s quality control standards. Follow-up started at first prescription for an antihypertensive drug and ended at the earliest of the following: 1) first break in antihypertensive therapy of >60 days; 2) death; 3) left practice; 4) last data collection from practice; or 5) diagnosis of end-stage renal disease (ESRD; Supplementary materials). We excluded patients with ESRD prior to study entry.

#### ACEI/ARB or other antihypertensive users

We calculated prescription duration using the quantity of medication prescribed and daily dose recorded; when these data were not available, we assumed the population median prescription duration (28 days). Exposure to medications was assumed to start on the date of the prescription. We constructed continuous courses of therapy by allowing for a 60-day gap between consecutive prescriptions (≤60 days between the end of one prescription and start of the next) to allow for stockpiling of drugs and nonadherence.

We classified individuals as ACEI/ARB users from their first ACEI/ARB prescription until their first break in continuous therapy of >60 days, regardless of concomitant β-blocker, CCB, or thiazide diuretic prescriptions. Individuals were identified as users of other antihypertensives (β-blockers, CCBs, or thiazide diuretics) from their first non-ACEI/ARB antihypertensive prescription until either their first ACEI/ARB prescription (at which point they were classified as ACEI/ARB users) or their first break in continuous antihypertensive therapy of >60 days. If a non-ACEI/ARB user did not have an AKI event during their first course of antihypertensive therapy with a specific drug class, they were considered for inclusion in the study in subsequent first courses of therapy with alternative drug classes (eg, if an individual had a new course of β-blocker therapy during which no AKI event occurred, they could be included if they had an AKI event during a subsequent new course of CCB therapy).

#### Exposure

We identified acute infections using morbidity coding in primary care (Read codes). We assumed records separated by ≤28 days represented the same episode of infection; the incident date of infection was taken to be the first in a series of consecutive records separated by ≤28 days.

Gastroenteritis was identified using an algorithm that employed the following types of code: 1) definite – codes that definitely represent gastrointestinal infection (eg, J43 – gastroenteritis); 2) symptom – codes that represent symptoms of gastroenteritis (eg, 19F – diarrhea); and 3) Pathogen – codes that represent specific microbiological causes of gastroenteritis (eg, A070 – *Escherichia coli* gastrointestinal tract infection). Gastroenteritis was defined using either of the following: 1) a single definite gastroenteritis code within the infection episode, recorded as part of a general practice consultation only (we excluded any records that might not represent a contemporaneous record of the patient’s condition; eg, we excluded records of hospital letters as their content may represent a past condition). If there was a symptom code recorded in the preceding 28 days (ie, earlier in the infection episode), the infection was assumed to have started on the earliest date the symptom was recorded within the infection episode; or 2) a combination of a symptom code followed by a record of a pathogen code in the subsequent 28 days. The symptom code must have been recorded as part of a general practice consultation (ie, not part of a letter).

Due to the absence of pathogen codes for UTI or LRTI, these infection episodes were therefore defined using diagnostic Read codes for these infections, with the first of these codes (again recorded as a general practice consultation, ie, excluding hospital letters) indicating an incident infection.

#### Outcome

We defined AKI as the first AKI International Classification of Diseases Version 10 (ICD-10) morbidity code recorded in an inpatient episode that started within 7 days of the start of a hospital admission (HES data) using N17 and N19 codes recorded in any diagnostic position. The SCCS method requires that multiple outcomes be independent of one another;^14^ because having one AKI event may alter the probability of having a subsequent AKI event, this assumption does not hold; therefore, we applied the established method of analyzing only the first AKI event for each patient.^14^

#### Comorbidities and demographics

For descriptive purposes, we identified sex, preexisting diabetes mellitus, ischemic heart disease, cardiac failure, hypertension, arrhythmia, decreased renal function (estimated glomerular filtration rate [eGFR] <60 mL/min/1.73 m^2^), the number of episodes of each type of infection during follow-up, and the number of AKI hospital admissions during follow-up. Diabetes mellitus, ischemic heart disease, cardiac failure, hypertension, and arrhythmia were identified using primary care and in-hospital morbidity coding prior to study entry. Renal function was established by calculating eGFR using the Chronic Kidney Disease Epidemiology Collaboration (CKD-EPI) equation.^19^ We used serum creatinine results recorded in the 12 months before first ACEI/ARB or other antihypertensive prescription to calculate eGFR (using either the highest eGFR from the most recent two serum creatinine results or, if only one creatinine result was available, the single most recent serum creatinine recorded prior to first prescription).

Current age was derived from date of birth and was included in all analyses as a time-varying covariate in the following age bands: 18–44, 45–54, 55–59, 60–64, 65–69, 70–74, 75–84, 85–89, and 90+ years. Time-varying diabetes mellitus, cardiac failure and loop diuretic exposure, and baseline renal function were included in secondary analyses. Diabetes and cardiac failure were defined as time-updated variables representing “ever diagnosed,” with status changing with the first-recorded code for each condition. Loop diuretic exposure was identified using continuous courses of loop therapy defined allowing for a 60-day gap between consecutive prescriptions. Baseline renal function was classified as eGFR above or below 60 mL/min/1.73 m.^2^

We have made code lists for all variables available for download (https://doi.org/10.17037/DATA.211).

### Statistical analysis

SCCS methodology is based on that used in cohort studies. It relies on making comparisons within individuals in a population of people who experience both the outcome and the exposure. Incidence rate ratios (IRRs) are calculated using conditional Poisson regression to compare the rate of events during exposed periods to rates observed during unexposed periods.^14^ This removes all fixed between-person confounding, ie, differences between people who did and did not have infections (such as comorbidities and risk factors for renal disease).

**Figure 1:**
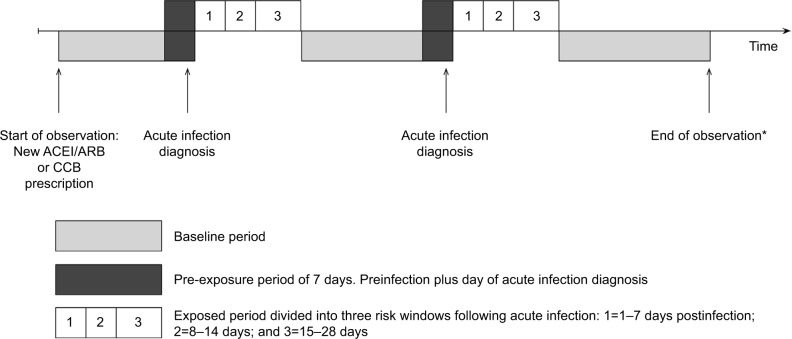
Graphical representation of self-controlled case series study design. **Notes:** Figure illustrates a single individual with an acute infection (UTI, LRTI, or gastroenteritis) during their observation period. All participants included in the analyses had at least one acute infection and at least one episode of AKI requiring hospital admission (analyses used first episode of AKI as the outcome and ignored subsequent AKI records). Rate ratios presented are pooled estimates derived from the rate of AKI events during risk (exposed) periods divided by the rate of events during baseline periods; age is adjusted for at all stages of analysis. Incident AKI can occur during any one of six exposure periods: baseline, 7 days prior to infection, day of infection, 1–7 days postinfection, 8–14 days postinfection, or 15–28 days postinfection. *Follow-up ends at the earliest of death, the end of registration, last collection date from GP, or 30/60/90 days after the end of first break in ACEI/ARB or CCB treatment of 30/60/90 days or more. **Abbreviations:** ACEI/ARB, angiotensin-converting enzyme inhibitor/angiotensin receptor blocker; AKI, acute kidney injury; CCB, calcium channel blocker; GP, general practitioner; LRTI, lower respiratory tract infection; UTI, urinary tract infection.

We defined a 1-month exposed period starting the day after infection diagnosis and subdivided it into three risk windows: 1–7, 8–14, and 15–28 days postinfection. All other observation times made up the baseline (unexposed) period, with the exception of the day of and the 7 days before infection diagnosis. We separated the 7 days prior to infection diagnosis from baseline time because infections may begin earlier than the date when an individual presents to their GP; therefore, during this time, they may be at a different risk of AKI, leading to possible bias in the baseline rate of AKI. Events recorded on the same day may have occurred on different days sometime prior to the GP visit but are recorded on the day of the consultation; we therefore also separated out the day of infection diagnosis from other follow-up time. Figure 1 illustrates the observation period for a single individual.

For each infection, we calculated the IRR comparing the rate of AKI in each risk window to baseline time using conditional Poisson regression and adjusting for age.

### Sensitivity analyses

We tested the impact of our AKI definition by repeating the main analysis: 1) limiting the defining ICD-10 codes to N17 codes only, which have been shown to have a high positive predictive value for AKI;^20^ and 2) restricting to codes recorded in the top two diagnostic positions of any hospital episode starting within 7 days of admission. We also varied the period over which we assumed continued antihypertensive exposure, allowing periods of 30 or 90 days between consecutive prescriptions. In addition, we repeated the main analysis including calendar period as a covariate to adjust for the many changes in clinical, diagnostic, and administrative practices over the study period that may influence number of reported AKI cases and the recording of incident infections (using the following time periods: 1997–2000, 2001–2004, 2005–2008, 2009–2011, and 2012–2014). Finally, the SCCS method requires that each individual’s observation period is independent of the outcome, an assumption that could be violated by fatal AKI events. Therefore, we excluded those who died within 90 days of their AKI event to assess the effect of censoring due to death.

### Secondary analyses

We undertook stratified analyses to explore whether the risk of AKI during periods of acute infection differed in the following groups: 1) ACEI/ARB users compared with other anti-hypertensive users; 2) those with and without cardiac failure and diabetes mellitus; 3) those with low baseline eGFR (<60 mL/min/1.73 m^2^); and 4) the use of a loop diuretic. In each instance, we calculated IRRs (adjusted for age) stratified by each binary variable using a model including an interaction between risk period and the stratifying variable and *p*-values (from likelihood ratio tests) to investigate whether the interaction explained more of the variability in AKI during risk periods compared with baseline time.

Post hoc we were concerned that our comparison group of users of non-ACEI/ARB antihypertensive drugs (β-blockers/CCBs/thiazides) was heterogeneous and likely to include many frail patients at a high risk of infection and AKI. Therefore, we repeated the analysis comparing ACEI/ARB users to users of CCBs only. Initially, CCB users were defined in the same way as that used in the main study population (refer to the section “ACEI/ARB or other-antihypertensive users”). We then repeated the analysis again, but this time included only follow-up time from the first study drug prescribed (ACEI/ARB or CCB) and censored at any change in study drug prescribing (eg, change from one class of study drug to the other or addition of other study drug).

Data management and analyses were performed using Stata Version 14 (StataCorp LP, College Station, TX, USA).

### Ethical approval

This study was approved by the London School of Hygiene and Tropical Medicine Research Ethics Committee (Reference 6536) and by the CPRD Independent Scientific Advisory Committee (ISAC; Protocol Number: 15_146).

## Results

**Figure 2:**
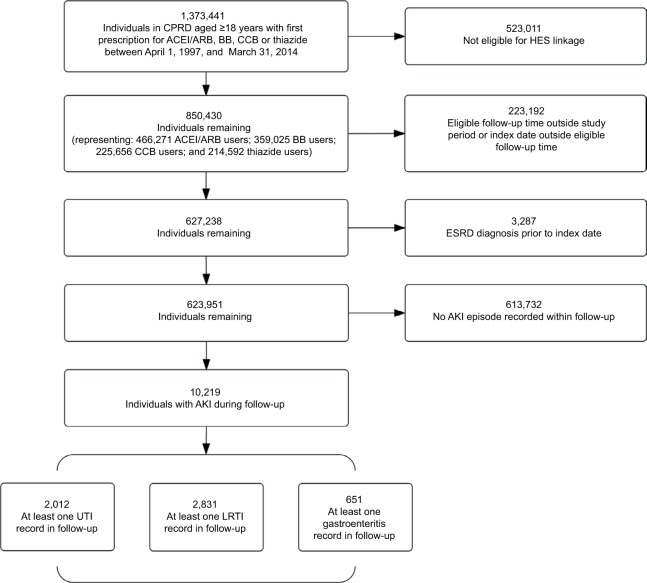
Identification of study participants. **Note:** UTI, LRTI, and gastroenteritis are analyzed as separate outcomes. **Abbreviations:** ACEI/ARB, angiotensin-converting enzyme inhibitor/angiotensin receptor blocker; AKI, acute kidney injury; BB, β-blocker; CCB, calcium channel blocker; CPRD, Clinical Research Practice Datalink; ESRD, end-stage renal disease; HES, Hospital Episode Statistics; LRTI, lower respiratory tract infection; UTI, urinary tract infection.

From a cohort of 623,951 eligible new users of antihypertensives, we identified 10,219 individuals with a record of AKI in the CPRD database (Figure 2). Among these, 2,012 had at least one record for a UTI during follow-up, 2,831 had a record for an LRTI, and 651 had a record for gastroenteritis. The population was elderly with a mean baseline age of 70–75 years and a high prevalence of comorbidities (Table 1). ACEI/ARB users were more likely than users of other antihypertensives to have comorbidities (diabetes mellitus, ischemic heart disease, arrhythmia, hypertension, and cardiac failure) at first antihypertensive prescription.

**Figure 3:**
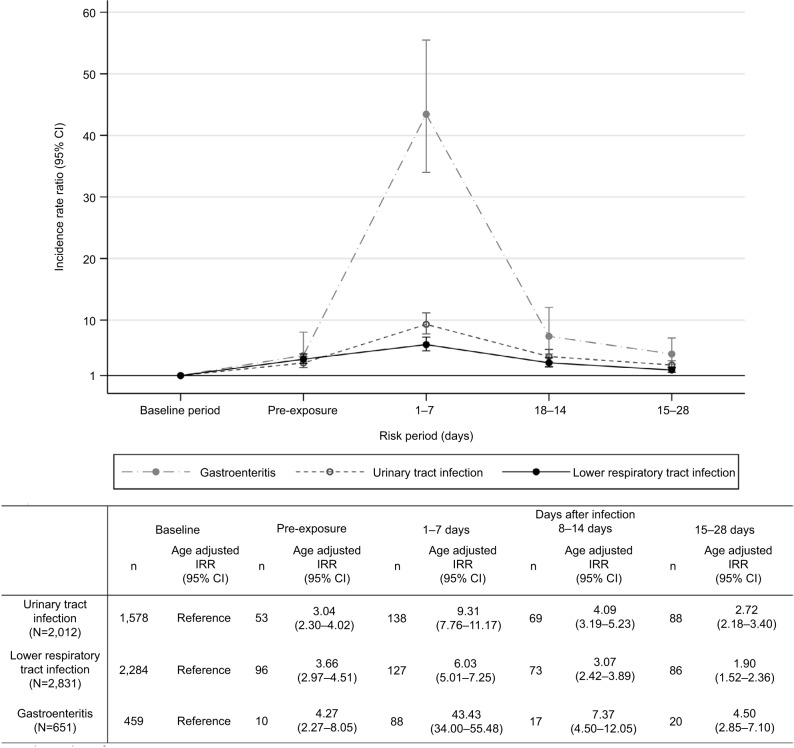
Main analysis: age-adjusted incidence rate ratios (95% CI) for AKI in risk periods after acute community-acquired infections (gastroenteritis, urinary tract infection, and lower respiratory tract infection). **Notes:** The numbers of participants exposed to each type of infection are shown in parentheses for each exposure. These include a small number who had a recorded AKI event on the day of infection exposure that was not included in the analysis, because the events may have been recorded retrospectively. Incidence during the baseline period served as the reference category. IRR denotes age-adjusted incidence ratio; age-adjusted in the following age bands: 18–44, 45–54, 55–59, 60–64, 65–69, 70–74, 75–84, 85–89, and 90+ years. Participants may appear within more than one category. **Abbreviations:** AKI, acute kidney injury; IRR, incidence rate ratio.

AKI risk was higher following infection than in baseline noninfectious periods (Figure 3). AKI rate ratio was highest following gastroenteritis; for the period 1–7 days postinfection, IRR for gastroenteritis was 43.3 (95% CI=34.0–55.5), compared with 6.0 in LRTI (95% CI=5.0–7.3) and 9.3 in UTI (95% CI=7.8–11.2). In the 7 days prior to infection, relative AKI risk was between 3 and 4 times higher (depending on infection type) than during baseline time, and AKI risk decreased over time following infection.

Changing the definition of AKI, allowing periods of 30 or 90 days (instead of 60) between consecutive prescriptions to define eligible follow-up time, additionally including calendar period as a covariate or excluding those who died within 90 days of their AKI event made minimal difference to the results (Table S1).

For LRTI and gastroenteritis, stratifying on the class of antihypertensive prescribed at baseline revealed no evidence of a difference in AKI risk in ACEI/ARB users compared with users of other antihypertensives (*p*-values for interaction: LRTI, *p*=0.784; gastroenteritis, *p*=0.879). For example, in the in the 1–7 days following LRTI, the IRR for AKI in non-ACEI/ARB users was 6.3 (95% CI=4.3–9.3) compared with 6.0 (95% CI=4.8–7.3) in ACEI/ARB users, while following gastroenteritis the IRR for AKI in non-ACEI/ARB users was 35.4 (95% CI=19.2–65.3) compared with45.2 (95% CI=34.6–59.1) in ACEI/ARB users (Table S2; Figure S1). There was little difference in effect estimates if we varied the definition used to identify non-ACEI/ARB users (Table S3).

There was some limited evidence of a difference in AKI risk in ACEI/ARB users compared with users of other antihypertensives following UTI (*p*-value for interaction: *p*=0.021). In the 1–7 days following UTI, the IRR for AKI in non-ACEI/ARB users was 7.4 (95% CI=5.1–10.7), compared with 10.0 (95% CI=8.2–12.4) in ACEI/ARB users (Table S2). However, there was no evidence for a difference in AKI risk between ACEI/ARB and non-ACEI/ARB users following UTI after varying the definition used to identify non-ACEI/ARB users (*p*-values for interaction: *p*=0.276 after defining non-ACEI/ARB users as CCB users; *p*=0.558 after defining non-ACEI/ARB users as CCB users and only including follow-up for the first drug prescribed [either ACEI/ARB or CCB]; Table S3).

After stratifying on cardiac failure, diabetes, loop diuretic exposure, and impaired baseline renal function (eGFR<60 mL/min/1.73 m^2^), there was some evidence of a difference in AKI risk in the sicker compared with the healthier subgroups in the first week of infection (Table S2); with some exceptions, the relative risk of AKI was lower in sicker subgroups. For example, 1) compared with nondiabetics, AKI risk was lower in diabetics in the week following LRTI or gastroenteritis, but higher in diabetics following UTI; 2) AKI risk was lower in those with cardiac failure than those without following any UTI, LRTI, or gastroenteritis; 3) following LRTI or gastroenteritis, the risk of AKI was lower in those on loop diuretics compared with nonloop users; and 4) following gastroenteritis, the risk of AKI was lower in those with worse renal function.

## Discussion

Our results show a substantially increased risk of hospital admission with AKI following periods of acute infection in the community. While this is widely believed to be the case, to our knowledge, this is the first study to quantify this risk. The risk of AKI was 43 times higher in the week following gastroenteritis compared with baseline time, but in addition, it was also substantially increased after UTI and LRTI. The magnitude of the increased risk of AKI following acute infection was similar for users of ACEI/ARBs or other antihypertensives.

### Strengths and weaknesses

A major strength of our study is the use of the SCCS design. Because this design compares risk within individuals at different times, results are less influenced by confounding from differences between comparison groups than traditional cohort designs. Therefore, differences in the prevalence of shared causes of infection and AKI risk, such as diabetes or other comorbidities, between the antihypertensive classes should not explain our results. Further, our study used routine, prospectively collected clinical data from a large UK general practice database that is broadly representative of the UK population.^15^ Our results, therefore, reflect real-world clinical practice and are likely to be generalizable. The baseline characteristics of the study population demonstrate that they are elderly with a high prevalence of comorbidities and so are representative of those known to be at a high overall risk of AKI. However, we studied only people taking antihypertensives, and it is possible that the risk of AKI after infections is different in healthier people not taking these medications.

We examined only three of the commonest infections (UTI, LRTI, and gastroenteritis)^21^–^24^ and so cannot comment on the risks of AKI following other infections. We used ICD-10 coding alone to define AKI; therefore, we have captured only a small proportion of the cases defined by current biochemical definitions of AKI. However, the vast majority of cases were identified using a code that has high positive predictive value for AKI and includes a greater proportion of more severe cases.^25^,^26^ While AKI coding patterns have changed markedly over recent years,^27^ adjusting for calendar period, or restricting to the top two diagnostic code positions, did not affect our results. We examined only the risk associated with patients’ first episode of AKI; therefore, these results may not be representative of risks for patients with recurrent AKI admissions.

It is possible that patients who subsequently developed AKI had features of sepsis when they visited their GP with infection. However, clinically evident sepsis is unlikely to explain our results since immediate hospital admission was not arranged (we separated out individuals with hospital AKI recorded on the same day as their primary care infection record). Our assessment of drug exposure was based on prescriptions alone. We cannot be certain that those prescribed a drug were taking the medication; crucially we were unable to capture any temporary discontinuation in medication use during acute illness. However, advice to withhold ACEIs/ARBs during acute illness (sick-day rules) is recent, and we did not see any change in our results after adjusting for calendar period. We made assumptions about likely exposure status for all drugs during apparent gaps in prescribing records. We chose to consider gaps of ≤60 days as indicating continued treatment, but alternative assumptions about the continuous treatment period had no substantial effect on our results.

Finally, there may be systematic differences between ACEI/ARB users and patients taking other antihypertensives. To make our results generalizable, patients taking ACEI/ARB were classified into this group regardless of other antihypertensive use. This group had a higher prevalence of comorbidities and were likely to have been taking more drugs overall than users of other antihypertensives. In addition, to minimize differences between groups, we allowed users of other antihypertensives to subsequently become ACEI/ARB users, but (as ACEI/ARB cessation may be associated with increased frailty and AKI risk^28^) we did not allow those who stopped the drugs to be classed as users of other antihypertensives. ACEI/ARB users were therefore followed up for longer, with more time to develop further comorbidities (which are themselves risk factors for AKI) or to become increasingly frail. A sensitivity analysis to examine the impact of this, restricting to follow-up time from first study drug prescribed (ACEI/ARB or CCB) to any change in study drug prescribing, showed little difference in effect estimates. Nonetheless, these differences between the groups mean that it is likely that those using ACEI/ARB had a higher absolute rate of AKI in all time periods. This could have resulted in a lower IRR for AKI following infections in ACEI/ARB users compared with the risk ratio for users of other antihypertensives.

### Results in context

Among critically ill patients, there is a clearly established causal link between sepsis and AKI^29^–^35^ with approximately half of AKI cases related to sepsis.^29^,^30^,^32^,^35^ AKI has also been shown to be common in those with less severe infections.^36^,^37^ There is evidence that in intensive care patients with AKI the most common source of sepsis is the lung.^31^,^32^,^34^

Our study design can only investigate the relative effect on AKI risk of each infection. However, the absolute rates of AKI and therefore public health impact depend on the incidence of each infection. LRTIs are the leading infection-related cause of disability-adjusted life years in England^38^ and lead to more hospitalizations than UTIs or gastroenteritis;^39^ therefore, while the relative risk of AKI following gastroenteritis may be higher than that following LRTI, LRTI may be associated with more AKI events than gastroenteritis or UTI. Similarly, UTIs are common in young women and those with diabetes,^40^ therefore, UTIs may be related to more AKI events in these groups than LRTIs or gastroenteritis.

### Explanations and implications

We found that, compared with baseline noninfected time, patients with gastroenteritis had a much higher risk of AKI following infection than those with UTI or LRTI. This is likely to relate to the differences in physiological disturbance caused by the different infections, with substantial volume depletion following gastroenteritis. In addition, there may be differences in primary care consulting behavior, or subsequent detection bias, meaning that patients with diarrhea are more likely to have diagnosed or recorded AKI compared with patients with UTI or LRTI.

These data also demonstrate some counterintuitive results. Compared with baseline noninfected time, we found that the relative risk of AKI was higher in the 7 days prior to infection diagnosis. This may have occurred if a proportion of patients were admitted directly to hospital with AKI and then consulted their GP in the week following their hospital admission for related or new infection symptoms. This would have led to capture of a hospital AKI record followed by a primary care infection code. If the initial hospital admission was related to infection, we would underestimate the association of infection with AKI in our results.

Our results also showed that the relative risk of AKI in the first week following infection compared with baseline noninfected time was, in general, slightly lower in predefined groups at a higher risk of AKI: cardiac failure, diabetes, additional loop diuretic exposure, and impaired baseline renal function.^28^ As discussed above, a lower degree of increased AKI risk after infection might be explained by patients with these chronic health conditions being more likely to have admissions for AKI during baseline time. Alternately, sicker patients might have been more likely to be sent to hospital by their GP on the day of their infection diagnosis or to bypass their GP by going straight to hospital. Since we separated out the day of infection diagnosis from other follow-up time and individuals without a primary care infection diagnosis were excluded from the study, we potentially systematically excluded sicker individuals both from postinfection risk periods and from inclusion in our study. Both explanations would again suggest that our estimates of the increase in the risk of AKI following infection are likely to be conservative. However, while we found that the relative impact of infection on AKI risk was, in general, slightly lower in sicker groups, those in subgroups with these comorbidities will be at higher absolute baseline risk of AKI; therefore, any relative increase will have proportionally more impact.

While our results showed no evidence of effect modification by ACEI/ARB use in the risk of AKI following LRTI (the analysis with the greatest number of events) or gastroenteritis, we did find statistical evidence of effect modification by ACEI/ARB use for AKI following UTI. However, differences in the effect estimates between ACEI/ARB users and those of other antihypertensives at each time point were small. After limiting the comparison group with those on CCBs only there was no longer any evidence for a difference in AKI risk between ACEI/ARB and non-ACEI/ARB users following UTI. In addition, given the multiple comparisons undertaken, we do not believe that our results demonstrate any evidence that people on ACEI/ARBs have a greater increase in the risk of AKI following infection than those on other antihypertensives. However, it is possible that due to differences between people taking different antihypertensives we have failed to detect a true difference. Nonetheless, if ACEI/ARB use was a strong risk factor for AKI after infection, we might have expected to see evidence of an interaction between antihypertensive group and the level of increased risk of AKI, as we have done for other known risk factors.

Current guidelines for health care professionals recommend that patients with hypovolemia, sepsis, and possible urological obstruction and those taking potentially nephrotoxic drugs should be considered to be a particular risk of AKI.^8^ Similarly, self-management guidelines for patients taking ACEI/ARBs, nonsteroidal anti-inflammatory drugs, diuretics, or metformin advise patients to stop taking these medications if they develop vomiting, diarrhea, or “fever, sweats, and shaking.”^41^ Our results confirm the need to emphasize gastroenteritis as a potent risk factor for AKI. However, they also suggest that current guidance should be broadened to include other types of infection. In addition, our results support previous research that patient characteristics are a much more important risk factor for AKI than specific classes of antihypertensive drugs.

## Conclusion

In conclusion, among antihypertensive users in primary care, UTI, LRTI, and gastroenteritis are all associated with a substantially increased risk of hospital admission with AKI. The level of increased risk is similar for both ACEI/ARB users and users of other antihypertensives. Clinicians should be aware of the increased risk of AKI in anyone with symptoms of these infections.

## Data sharing statement

No additional unpublished data are available as this study used existing data from the UK CPRD and HES electronic health record databases that are accessible to researchers following protocol approval by the CPRD’s ISAC.

## Supplementary materials

Figure S1 Age-adjusted incidence rate ratios (95% CI) for AKI in risk periods after community-acquired infections stratified by ACEI/ARB or other antihypertensive use.
**Note:** Other antihypertensives: β-blockers, calcium channel blockers, or thiazide diuretics.
**Abbreviations:** ACEI/ARB, angiotensin-converting enzyme inhibitor/angiotensin receptor blocker; AKI, acute kidney injury.

**Table S1:** Age-adjusted incidence rate ratios (95% CIs) for AKI during risk periods compared with baseline time in the main analysis and additional sensitivity analyses

	Risk period	Urinary tract infection	Lower respiratory tract infection	Gastroenteritis
Main analysis: AKI defined using N17 and N19 codes in any diagnostic position in any episode starting within 7 days of hospital admission; 60-day follow-up definition ^a^		n=2,012	n=2,831	n=651
	Baseline period	Reference	Reference	Reference
	Days after infection:			
	1–7	9.31 (7.76, 11.17)	6.03 (5.01, 7.25)	43.43 (34.00, 55.48)
	8–14	4.09 (3.19, 5.23)	3.07 (2.42, 3.89)	7.37 (4.50, 12.05)
	15–28	2.72 (2.18, 3.40)	1.90 (1.52, 2.36)	4.50 (2.85, 7.10)
ICD-10 N17 AKI: AKI defined using N17 codes only in any diagnostic position in any episode starting within 7 days of hospital admission; 60-day follow-up definition ^a^		n=1,583	n=2,154	n=531
	Baseline period	Reference	Reference	Reference
	Days after infection:			
	1–7	10.72 (8.79, 13.09)	6.84 (5.56, 8.41)	47.58 (36.32, 62.34)
	8–14	4.85 (3.72, 6.33)	3.32 (2.53, 4.36)	8.65 (5.11, 14.64)
	15–28	3.07 (2.40, 3.92)	2.05 (1.60, 2.64)	4.79 (2.87, 7.97)
Top diagnostic position: AKI defined using N17 and N19 codes recorded in the top two diagnostic positions in any episode starting within 7 days of hospital admission; 60-day follow-up definition ^a^		n=891	n=1,086	n=288
	Baseline period	Reference	Reference	Reference
	Days after infection:			
	1–7	10.42 (8.02, 13.53)	5.98 (4.42, 8.08)	62.81 (44.01, 89.65)
	8–14	4.18 (2.89, 6.04)	3.18 (2.18, 4.65)	11.14 (5.62, 22.07)
	15–28	2.95 (2.14, 4.07)	2.15 (1.53, 3.01)	7.75 (4.25, 14.13)
30-day follow-up: follow-up ends at the earliest of death, the end of registration, last collection date from GP, or 30 days after end of first break in the treatment of ≥30 days (main analysis uses 60 days)		n=1,095	n=1,466	n=318
	Baseline period	Reference	Reference	Reference
	Days after infection:			
	1–7	8.26 (6.53, 10.45)	7.28 (5.82, 9.12)	53.12 (38.31, 73.65)
	8–14	3.59 (2.61, 4.94)	3.05 (2.23, 4.18)	12.49 (7.26, 21.48)
	15–28	2.43 (1.82, 3.22)	2.17 (1.65, 2.86)	6.03 (3.45, 10.53)
90-day follow-up: follow-up ends at the earliest of death, the end of registration, last collection date from GP, or 90 days after end of first break in the treatment of ≥90 days (main analysis uses 60 days)		n=2,444	n=3,468	n=805
	Baseline period	Reference	Reference	Reference
	Days after infection:			
	1–7	9.04 (7.62, 10.72)	5.78 (4.85, 6.87)	43.08 (34.36, 54.00)
	8–14	4.09 (3.26, 5.14)	2.95 (2.36, 3.68)	8.70 (5.69, 13.29)
	15–28	2.66 (2.16, 3.26)	1.86 (1.52, 2.28)	4.48 (2.93, 6.84)
Calendar period: Adjusted for calendar period in addition to age; ^b^ main AKI definition; 60-day follow-up definition ^a^		n=2,012	n=2,831	n=651
	Baseline period	Reference	Reference	Reference
	Days after infection:			
	1–7	8.46 (7.05, 10.15)	5.59 (4.64, 6.73)	41.72 (32.52, 53.52)
	8–14	3.71 (2.90, 4.75)	2.83 (2.23, 3.59)	7.06 (4.30, 11.58)
	15–28	2.47 (1.98, 3.08)	1.73 (1.38, 2.15)	4.27 (2.70, 6.76)
Excluding early deaths: excludes all those who died within 60 days of AKI event; main AKI definition; 60-day follow-up definition ^a^		n=1,542	n=1,965	n=516
	Baseline period	Reference	Reference	Reference
	Days after infection:			
	1–7	9.05 (7.31, 11.21)	5.25 (4.15, 6.64)	50.14 (38.36, 65.52)
	8–14	4.12 (3.09, 5.48)	2.50 (1.83, 3.41)	8.21 (4.77, 14.12)
	15–28	2.50 (1.92, 3.26)	1.38 (1.02, 1.87)	4.50 (2.66, 7.61)

**Notes:**

a Follow-up ends at the earliest of death, the end of registration, last collection date from GP, or 30/60/90 days after the end of first break in the treatment of 30/60/90 days or more. IRR denotes age-adjusted incidence ratio; age-adjusted in the following age bands: 18–44, 45–54, 55–59, 60–64, 65–69, 70–74, 75–84, 85–89, and 90+ years.

b Adjusted for calendar time using the following periods: 1997–2000, 2001–2004, 2005–2008, 2009–2011, and 2012–2014.

**Abbreviations:** AKI, acute kidney injury; GP, general practitioner; ICD-10, International Classification of Diseases Version 10; IRR, incidence rate ratio.

**Table S2:** Age-adjusted incidence rate ratios (95% CI) for AKI in risk periods after acute infections, stratified by ACEI/ARB or CCB exposure, time-updated diabetes mellitus, cardiac failure and loop diuretic exposure, and baseline renal function

	Urinary tract infection				Lower respiratory tract infection				Gastroenteritis			
	No. of cases	IRR (95% CI)	No. of cases	IRR (95% CI)	No. of cases	IRR (95% CI)	No. of cases	IRR (95% CI)	No. of cases	IRR (95% CI)	No. of cases	IRR (95% CI)
**Antihypertensive drug prescribed at baseline**	(n=2,012, *p* =0.021)				(n=2,831, *p*=0.784)				(n=651, *p* 0.879)			
	**Non-ACEI/ARB (n=470)**		**ACEI/ARB (n=1,542)**		**Non-ACEI/ARB (n=551)**		**ACEI/ARB (n=2,280)**		**Non-ACEI/ARB (n=108))**		**ACEI/ARB (n=543)**	
Baseline period	369	Reference	1,209	Reference	420	Reference	1,864	Reference	76	Reference	383	Reference
Days after infection:												
1–7	33	7.37 (5.06, 10.72)	105	10.04 (8.16, 12.37)	30	6.33 (4.30, 9.33)	97	5.95 (4.82, 7.34)	14	35.43 (19.21, 65.33)	74	45.22 (34.62, 59.07)
8–14	13	2.59 (1.47, 4.55)	56	4.68 (3.56, 6.17)	19	3.60 (2.24, 5.79)	54	2.92 (2.22, 3.85)	<5 ^a^	4.39 (1.06, 18.21)	15	8.07 (4.77, 13.64)
15–28	20	2.10 (1.32, 3.33)	68	2.96 (2.30, 3.81)	17	1.73 (1.05, 2.85)	69	1.94 (1.52, 2.49)	<5 ^a^	4.80 (1.72, 13.40)	16	4.43 (2.66, 7.37)

**Diabetes mellitus**	(n=2,012, *p* <0.0001)				(n=2,831, *p*<0.0001)				(n=651, *p* <0.0001)			
	**No DM (n=1,251)**		**DM (n=761)**		**No DM (n=1,815)**		**DM (n=1,016)**		**No DM (n=385)**		**DM (n=266)**	
Baseline period	981	Reference	579	Reference	1,432	Reference	852	Reference	255	Reference	204	Reference
Days after infection:												
1–7	85	9.03 (7.16, 11.38)	53	9.87 (7.35, 13.25)	88	6.25 (5.00, 7.81)	39	5.69 (4.09, 7.93)	59	50.32 (37.08, 68.30)	29	35.91 (23.66, 54.52)
8–14	44	4.11 (3.02, 5.61)	25	4.07 (2.70, 6.13)	55	3.48 (2.64, 4.58)	18	2.29 (1.43, 3.68)	<5 ^a^	10.44 (6.02, 18.09)	<5 ^a^	3.29 (1.04, 10.39)
15–28	45	2.20 (1.62, 2.98)	43	3.63 (2.63, 5.01)	58	1.93 (1.48, 2.53)	28	1.85 (1.26, 2.71)	12	4.66 (2.59, 8.41)	8	4.33 (2.10, 8.94)

**Cardiac failure**	(n=2,012, *p* <0.0001)				(n=2,831, *p*<0.0001)				(n=651, *p* <0.0001)			
	**No CF (n=1,362)**		**CF (n=650)**		**No CF (n=1,653)**		**CF (n=1,178)**		**No CF (n=456)**		**CF (n=195)**	
Baseline period	1,045	Reference	533	Reference	1,337	Reference	947	Reference	303	Reference	156	Reference
Days after infection:												
1–7	90	9.63 (7.69, 12.05)	48	9.61 (6.95, 13.28)	72	6.70 (5.25, 8.55)	55	5.16 (3.85, 6.92)	71	55.65 (42.15, 73.48)	17	23.40 (13.48, 40.63)
8–14	51	4.80 (3.60, 6.40)	18	3.13 (1.92, 5.10)	36	2.95 (2.11, 4.13)	37	2.95 (2.08, 4.17)	11	7.53 (4.09, 13.86)	6	7.06 (3.01, 16.54)
15–28	65	3.18 (2.46, 4.12)	23	1.98 (1.28, 3.08)	5	1.93 (1.43, 2.61)	41	1.63 (1.17, 2.27)	16	5.72 (3.43, 9.56)	<5 ^a^	2.22 (0.80, 6.19)

**Loop diuretic exposure**	(n=2,012, *p* =0.680)				(n=2,831, *p*<0.0001)				(n=651, *p* =0.053)			
	**Time unexposed (n=1,429)**		**Time exposed (n=583)**		**Time unexposed (n=1,890)**		**Time exposed (n=941)**		**Time unexposed (n=466)**		**Time exposed (n=185)**	
Baseline period	1,117	Reference	461	Reference	1,537	Reference	747	Reference	323	Reference	136	Reference
Days after infection:												
1–7	93	9.00 (7.22, 11.23)	45	9.98 (7.23, 13.79)	86	6.77 (5.41, 8.48)	41	4.88 (3.52, 6.76)	65	48.53 (36.43, 64.65)	23	32.09 (19.81, 51.99)
8–14	52	4.39 (3.30, 5.83)	27	3.37 (2.05, 5.53)	41	2.87 (2.09, 3.93)	32	3.36 (2.33, 4.83)	13	8.41 (4.78, 14.80)	<5 ^a^	5.05 (1.83, 13.90)
15–28	63	2.72 (2.09, 3.53)	25	2.72 (1.79, 4.12)	51	1.82 (1.37, 2.42)	35	2.00 (1.41, 2.83)	12	3.80 (2.11, 6.83)	8	5.89 (2.82, 12.30)

**Baseline renal function**	(n=1,337, *p*	=0.213)			(n=1,797,	*p*=0.942)			(n=427, *p*	=0.054)		
	**eGFR ≥60 (n=737)**		**eGFR <60 (n=600)**		**eGFR ≥60 (n=1,096)**		**eGFR <60 (n=701)**		**eGFR ≥60 (n=280)**		**eGFR <60 (n=147)**	
Baseline period	1,122	Reference	456	Reference	1,737	Reference	547	Reference	350	Reference	109	Reference
Days after infection:												
1–7	91	9.53 (7.62, 11.91)	47	8.84 (6.45, 12.12)	91	6.02 (4.84, 7.48)	36	6.05 (4.26, 8.59)	75	52.51 (40.16, 68.66)	13	20.40 (11.07, 37.60)
8–14	47	4.32 (3.20, 5.82)	22	3.65 (2.35, 5.65)	52	3.04 (2.29, 4.02)	21	3.15 (2.01, 4.92)	11	6.76 (3.68, 12.42)	6	8.25 (3.53, 19.27)
15–28	56	2.69 (2.04, 3.54)	32	2.76 (1.90, 3.99)	58	1.76 (1.35, 2.30)	28	2.24 (1.52, 3.32)	17	5.41 (3.29, 8.89)	<5 ^a^	2.17 (0.68, 6.96)

**Notes:** The numbers of participants exposed to each type of infection are shown in parentheses for each exposure (and for each stratum for stratifying variables). These include a small number who had a recorded AKI event on the day of infection exposure that was not included in the analysis, because the events may have been recorded retrospectively. Incidence during the baseline period served as the reference category. Note that the number of cases during each baseline time and each risk period will not add up to the total number of cases in each analysis, as events on the day of infection and the 7 days before are not included in this table. Results are from five separate regression models for each infection; each model contains an interaction between the exposure (baseline time or risk periods following infection) and the stratifying variable (eg, diabetes).

a Cell counts <5 suppressed. Age-adjusted in the following age bands: 18–44, 45–54, 55–59, 60–64, 65–69, 70–74, 75–84, 85–89, and 90+ years. All *p*-values are for interaction terms (from likelihood ratio tests comparing main analysis with analysis including interactions with one of the following: antihypertensive drugs prescribed at baseline, diabetes mellitus, cardiac failure, loop diuretic use, or baseline renal function; *p*-values in bold are <0.05.

**Abbreviations:** ACEI/ARB, angiotensin-converting enzyme inhibitor/angiotensin receptor blocker; AKI, acute kidney injury; CCB, calcium channel blocker; CF, cardiac failure; DM, diabetes mellitus; eGFR, estimated glomerular filtration rate; IRR, incidence rate ratio.

**Table S3:** Age-adjusted incidence rate ratios (95% CI) for AKI in risk periods after acute infections, stratified by ACEI/ARB and non-ACEI/ARB exposure – with main analysis study population and alternative study populations (based on alternative ACEI/ARB/non-ACEI/ARB exposure definitions)

	Main analysis study population (ACEI/ARB and BB/CCB/thiazide users) ^a^		Alternative study population 1 (CCB and ACEI/ARB users) ^b^		Alternative study population 2 (CCB and ACEI/ARB users) ^c^	
	Non-ACEI/ARB	ACEI/ARB	CCB	ACEI/ARB	CCB	ACEI/ARB
**Urinary tract infection**	(n=2,012, ***p*=0.021**)		(n=1,730, *p*=0.276)		(n=1,023, *p*=0.558)	
Baseline period	Reference	Reference	Reference	Reference	Reference	Reference
Days after infection:						
1–7	7.37 (5.06, 10.72)	10.04 (8.16, 12.37)	6.88 (3.81 to 12.42)	9.85 (7.98 to 12.15)	6.92 (3.84 to 12.49)	8.92 (6.73 to 11.83)
8–14	2.59 (1.47, 4.55)	4.68 (3.56, 6.17)	2.30 (0.93 to 5.69)	4.68 (3.56 to 6.16)	2.31 (0.93 to 5.72)	4.52 (3.15 to 6.47)
15–28	2.10 (1.32, 3.33)	2.96 (2.30, 3.81)	1.64 (0.75 to 3.57)	2.96 (2.30 to 3.81)	1.65 (0.76 to 3.58)	2.44 (1.71 to 3.48)

**Lower respiratory tract infection**	(n=2,831, *p*=0.784)		(n=2,515, *p*=0.636)		(n=1,464, *p*=0.480)	
Baseline period	Reference	Reference	Reference	Reference	Reference	Reference
Days after infection:						
1–7	6.33 (4.30, 9.33)	5.95 (4.82, 7.34)	8.19 (4.73 to 14.20)	5.94 (4.81 to 7.33)	8.22 (4.74 to 14.24)	6.36 (4.88 to 8.29)
8–14	3.60 (2.24, 5.79)	2.92 (2.22, 3.85)	4.32 (2.17 to 8.60)	2.92 (2.21 to 3.84)	4.33 (2.17 to 8.62)	2.67 (1.85 to 3.87)
15–28	1.73 (1.05, 2.85)	1.94 (1.52, 2.49)	1.96 (0.95 to 4.06)	1.97 (1.54 to 2.51)	1.97 (0.95 to 4.07)	2.02 (1.48 to 2.77)

**Gastroenteritis**	(n=651, *p*=0.879)		(n=573, *p*=0.434)		(n=295, *p*=0.727)	
Baseline period	Reference	Reference	Reference	Reference	Reference	Reference
Days after infection:						
1–7	35.43 (19.21, 65.33)	45.22 (34.62, 59.07)	64.02 (21.56 to 190.08)	45.13 (34.55 to 58.96)	63.50 (21.59 to 186.79)	46.46 (32.26 to 66.91)
8–14	4.39 (1.06, 18.21)	8.07 (4.77, 13.64)	10.97 (1.39 to 86.40)	8.05 (4.76 to 13.61)	10.89 (1.39 to 85.28)	8.25 (4.02 to 16.94)
15–28	4.80 (1.72, 13.40)	4.43 (2.66, 7.37)	11.69 (2.55 to 53.53)	4.41 (2.65 to 7.35)	11.60 (2.55 to 52.74)	6.89 (3.87 to 12.26)

**Notes:** The numbers of participants exposed to each type of infection are shown in parentheses for each exposure. These include a small number who had a recorded AKI event on the day of infection exposure that was not included in the analysis, because the events may have been recorded retrospectively. Incidence during the baseline period served as the reference category.

a Main analysis study population: individuals were classified as ACEI/ARB users from their first ACEI/ARB prescription until their first break in continuous therapy of >60 days, regardless of concomitant β-blocker, CCB, or thiazide diuretic prescriptions. Individuals were identified as users of other antihypertensives (β-blockers, CCBs, or thiazide diuretics) from their first non-ACEI/ARB antihypertensive prescription until either their first ACEI/ARB prescription (at which point they were classified as ACEI/ARB users) or their first break in continuous antihypertensive therapy of >60 days. If a non-ACEI/ARB user did not have an AKI event during their first course of antihypertensive therapy with a specific drug class, they were considered for inclusion in the study in subsequent first courses of therapy with alternative drug classes.

b Alternative study population 1 (CCB and ACEI/ARB users): individuals were classified as ACEI/ARB users from their first ACEI/ARB prescription until their first break in continuous therapy of >60 days, regardless of concomitant CCB prescriptions. Individuals were identified as CCB users from their first CCB prescription until either their first ACEI/ARB prescription (at which point they were classified as ACEI/ARB users) or their first break in continuous CCB therapy of >60 days.

c Alternative study population 2 (CCB and ACEI/ARB users): individuals were classified as ACEI/ARB or CCB users based on the first drug prescribed (those whose first prescription was for both drugs were excluded from this analysis) and were censored at any change in study drug prescribing (ie, change from one class of study drug to the other or addition of the other study drug). All *p*-values are for interaction terms (from likelihood ratio tests comparing main analysis with analysis including interaction with ACEI/ARB or CCB/non-ACEI/ARB use).

**Abbreviations:** ACEI/ARB, angiotensin converting enzyme inhibitor/angiotensin receptor blocker; AKI, acute kidney injury; BB, β-blocker; CCB, calcium channel blocker.

## Figures and Tables

**Table 1:** Characteristics of study populations (antihypertensive drug users who had both the infection listed in the column heading and an episode of acute kidney injury during hospital admission were included [for details, see Figure 2]).

	Urinary tract infection			Lower respiratory tract infection			Gastroenteritis		
	Study population	ACEI/ARB	Non-ACEI/ARB	Study population	ACEI/ARB	Non-ACEI/ARB	Study population	ACEI/ARB	Non-ACEI/ARB
	n=2,012	n=1,542	n=470	n=2,831	n=2,280	n=551	n=651	n=543	n=108
Median (IQR) duration of available data (years)	5.3 (2.6, 8.2)	5.5 (2.8, 8.3)	4.3 (2.0, 7.9)	5.4 (2.7, 8.2)	5.6 (2.9, 8.4)	4.2 (1.7, 7.3)	5.8 (3.2, 8.6)	5.8 (3.3, 8.8)	5.2 (2.7, 7.1)
Female (%)	1,107 (55.0)	836 (54.2)	271 (57.7)	1,211 (42.8)	953 (41.8)	258 (46.8)	329 (50.5)	259 (47.7)	70 (64.8)
Mean (SD) baseline age	73.6 (11.6)	73.2 (11.6)	75.1 (11.3)	72.2 (11.5)	71.8 (11.3)	73.9 (12.0)	70.0 (11.3)	69.8 (11.3)	71.1 (11.2)
Mean (SD) number of infections in follow-up^a^	1.91 (1.83)	1.92 (1.87)	1.90 (1.67)	2.07 (2.15)	2.12 (2.22)	1.88 (1.79)	1.09 (0.32)	1.09 (0.31)	1.12 (0.35)
Mean (SD) number of AKI hospital admissions in follow-up	1.34 (0.77)	1.35 (0.79)	1.31 (0.71)	1.34 (1.02)	1.36 (1.07)	1.26 (0.73)	1.31 (0.65)	1.33 (0.67)	1.21 (0.51)
Number of individuals (%) with >1 AKI episode in follow-up	314 (15.6)	250 (16.2)	64 (13.6)	421 (14.9)	361 (15.9)	60 (10.9)	108 (16.6)	94 (17.3)	14 (13.0)
**Baseline comorbidities**									
Diabetes mellitus (%)	584 (29.0)	509 (33.0)	75 (16.0)	757 (26.7)	681 (29.9)	76 (13.8)	201 (30.9)	190 (35.0)	11 (10.2)
Ischemic heart disease (%)	600 (29.8)	485 (31.5)	115 (24.5)	926 (32.7)	790 (34.6)	136 (24.7)	229 (35.2)	203 (37.4)	26 (24.1)
Arrhythmia (%)	360 (17.9)	282 (18.3)	78 (16.6)	532 (18.8)	426 (18.7)	106 (19.2)	105 (16.1)	86 (15.8)	19 (17.6)
Hypertension (%)	1,257 (62.5)	996 (64.6)	261 (55.5)	1,710 (60.4)	1,407 (61.7)	303 (55.0)	387 (59.4)	332 (61.1)	55 (50.9)
Cardiac failure (%)	286 (14.2)	261 (16.9)	25 (5.3)	543 (19.2)	494 (21.7)	49 (8.9)	87 (13.4)	84 (15.5)	<5 (<5)^c^
**Baseline renal function**^b^									
eGFR ≥60 (%)	737 (36.6)	614 (39.8)	123 (26.2)	1,096 (38.7)	940 (41.2)	156 (28.3)	280 (43.0)	240 (44.2)	40 (37.0)
eGFR <60 (%)	600 (29.8)	486 (31.5)	114 (24.3)	701 (24.8)	585 (25.7)	116 (21.1)	147 (22.6)	129 (23.8)	18 (16.7)
Missing (%)	675 (33.5)	442 (28.7)	233 (49.6)	1,034 (36.5)	755 (33.1)	279 (50.6)	224 (34.4)	174 (32.0)	50 (46.3)

**Notes:** Figures are n (%) unless otherwise stated.

a Mean urinary tract infection count under urinary tract infection column, etc.

b Renal function calculated using serum creatinine test results recorded in the 12 months before index antihypertensive (ACEI/ARB, β-blocker, CCB, or thiazide diuretic) prescription. Based on best of most recent two serum creatinine results before antihypertensive prescription or single result if only one recorded in 12 months before index prescription.

c Cell counts <5 suppressed.

**Abbreviations:** ACEI, angiotensin-converting enzyme inhibitor; ARB, angiotensin receptor blocker; CCB, calcium channel blocker; eGFR, estimated glomerular filtration rate; IRO, interquartile range.
