# Supplementary figures and images for: Acute kidney injury and infections in patients taking antihypertensive drugs: a self-controlled case series analysis
Source: Clin Epidemiol. 2018 Jan 30;10:187–202. doi: 10.2147/CLEP.S146757 (PMC5796801; doi:10.2147/CLEP.S146757)

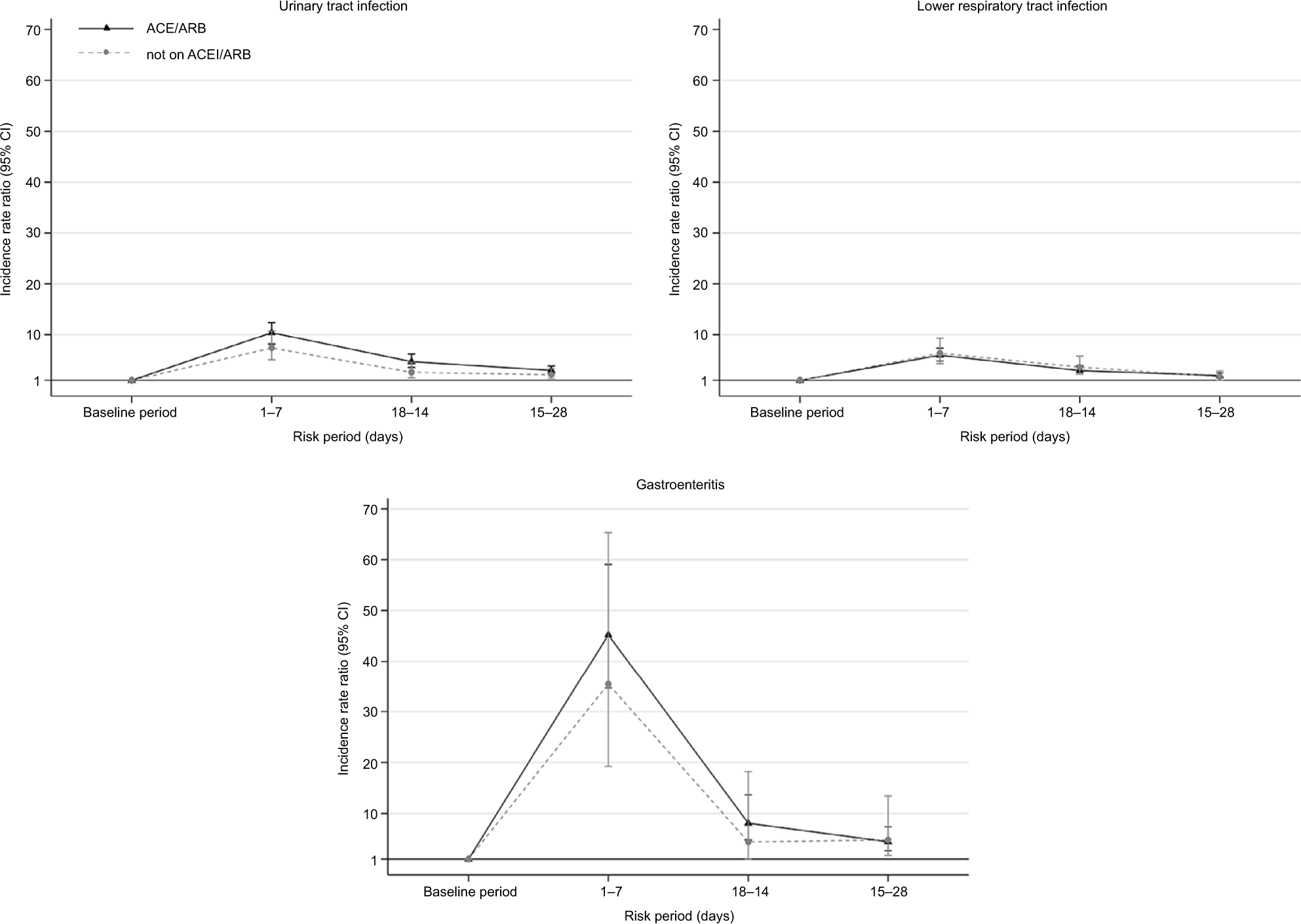

Supplement: Figure S1 — Age-adjusted incidence rate ratios (95% CI) for AKI in risk periods after community-acquired infections stratified by ACEI/ARB or other antihypertensive use. Note: Other antihypertensives: β-blockers, calcium channel blockers, or thiazide diuretics. Abbreviations: ACEI/ARB, angiotensin-converting enzyme inhibitor/angiotensin receptor blocker; AKI, acute kidney injury. [file clep-10-187s1.tif]
